# Diagnosis of Fanconi Anemia: Mutation Analysis by Multiplex Ligation-Dependent Probe Amplification and PCR-Based Sanger Sequencing

**DOI:** 10.1155/2012/603253

**Published:** 2012-06-21

**Authors:** Johan J. P. Gille, Karijn Floor, Lianne Kerkhoven, Najim Ameziane, Hans Joenje, Johan P. de Winter

**Affiliations:** Department of Clinical Genetics, VU University Medical Center, Van der Boechorsttraat 7, 1081 BT Amsterdam, The Netherlands

## Abstract

Fanconi anemia (FA) is a rare inherited disease characterized by developmental defects, short stature, bone marrow failure, and a high risk of malignancies. FA is heterogeneous: 15 genetic subtypes have been distinguished so far. A clinical diagnosis of FA needs to be confirmed by testing cells for sensitivity to cross-linking agents in a chromosomal breakage test. As a second step, DNA testing can be employed to elucidate the genetic subtype of the patient and to identify the familial mutations. This knowledge allows preimplantation genetic diagnosis (PGD) and enables prenatal DNA testing in future pregnancies. Although simultaneous testing of all FA genes by next generation sequencing will be possible in the near future, this technique will not be available immediately for all laboratories. In addition, in populations with strong founder mutations, a limited test using Sanger sequencing and MLPA will be a cost-effective alternative. We describe a strategy and optimized conditions for the screening of *FANCA, FANCB, FANCC, FANCE, FANCF,* and *FANCG* and present the results obtained in a cohort of 54 patients referred to our diagnostic service since 2008. In addition, the follow up with respect to genetic counseling and carrier screening in the families is discussed.

## 1. Introduction

Fanconi anemia (FA) is a rare inherited syndrome with diverse clinical symptoms including developmental defects, short stature, bone marrow failure, and a high risk of malignancies. Fifteen genetic subtypes have been distinguished: FA-A, -B, -C, -D1, -D2, -E, -F, -G, -I, -J, -L, -M, -N, -O, and -P. [1–4]. The majority of patients (~85%) belong to the subtypes A (~60%), C (~10–15%), or G (~10%), while a minority (~15%) is distributed over the remaining 12 subtypes, with relative prevalences between <1 and 5%. These percentages may differ considerably within certain ethnic groups, due to founder effects. All subtypes seem to fit within a “classical” FA phenotype, except for D1 and N (mutated in *BRCA2/FANCD1 *and *PALB2/FANCN*), which are associated with a distinct, more severe, syndromic association. The mode of inheritance for all subtypes is autosomal recessive, except for FA-B, which is X-linked. These two different modes of inheritance have important consequences for the counseling of FA families. The relative prevalence of FA-B amongst unselected FA patients is estimated at 1.6% [5]. For all genetic subtypes disease genes have been identified (Table 1). Many mutations found in the various subtypes are private, but recurrent mutations are known, particularly in specific ethnic backgrounds (Table 2).

Most FA genes encode orphan proteins with no known molecular function. At least eight FA proteins (FANCA, -B, -C, -E, -F, -G, -L, and -M) assemble into a nuclear multiprotein core complex, which is required to activate FANCD2 and FANCI by monoubiquitination [6]. FANCL, which carries a RING finger domain, is supposed to represent the ubiquitin E3 ligase in this activation [7]. FANCM probably acts as a sensor of DNA damage and recruits the FA core complex to the site of damage, but FANCM also interacts with other proteins including Blm [6]. Monoubiquitination of FANCD2 and FANCI directs these proteins to areas of damaged chromatin where they interact with other proteins, resulting in repair of the damage [6]. The remaining FA proteins function downstream of or parallel to the FANCD2 activation step [6]. The exact nature of the DNA damage response, which when defective causes FA, remains to be defined. FANCJ/BRIP1 and FANCM possess DNA helicase motifs, which strongly suggests that the FA pathway acts through a direct interaction with DNA, presumably to resolve or remodel blocked DNA replication forks resulting from DNA interstrand cross-link damage [6]. This idea is strengthened by the recent extension of the FA pathway with SLX4, a scaffold protein for structure-specific endonucleases involved in unhooking the DNA cross-link [3, 4].

## 2. Laboratory Diagnostics in FA

Cells derived from FA patients are—by definition—hypersensitive to chromosomal breakage induced by DNA cross-linking agents such as mitomycin C (MMC) or diepoxybutane (DEB) [31]. This cellular phenotype is ascertained using stimulated blood T lymphocytes. The indications for FA laboratory testing are rather broad [32]. As a consequence, in only a small proportion of patients (about 10%) the chromosomal breakage test is positive, and an FA diagnosis is established. Since mutation testing by Sanger sequencing and MLPA is rather laborious, time consuming and therefore expensive, a positive chromosomal breakage test is a prerequisite for starting mutation screening. Confirmation of the FA diagnosis at the DNA level is important in patients in whom the chromosomal breakage test was inconclusive. Furthermore, knowledge about the FA subtype is relevant for the treatment and prognosis of the patients. In addition, identification of mutations allows carrier testing in the family and will enable prenatal DNA testing and preimplantation genetic diagnosis (PGD) in future pregnancies. Finally, this information can be used to rule out FA in potential donors for bone marrow transplantation.

Although simultaneous testing of all FA genes by next generation sequencing will be possible in the near future, this technique will not be available immediately for all laboratories worldwide. In addition, in populations with strong founder mutations, a limited test using Sanger sequencing and MLPA will be a cost-effective alternative [33]. The strategy outlined below has been developed at our DNA diagnostics laboratory to provide a molecular diagnosis of FA. It is recognized that mutations in *FANCA* account for 60–70% of all FA cases and that about 15–20% of the mutations in this gene are large deletions [33, 34]. Therefore, DNA testing usually starts with a screen for deletions in *FANCA*. However, depending on the circumstances strategies may differ from case to case.

### 2.1. Materials

Genomic DNA (from e.g., leukocytes or fibroblasts derived from the proband or the parents) is adequate for most mutation screening assays. Screening on cDNA is more efficient but has several drawbacks: for high-quality cDNA, growing cells (stimulated leukocytes, lymphoblastoid cell lines, or fibroblasts) are necessary. In addition, common alternative splice variants will hamper the evaluation of DNA sequences. Therefore, screening on gDNA is the preferred method for mutation screening. However, during the diagnostic process, growing cells from the proband will be helpful in a couple of situations. Growing cells are indispensable for studying the effect of unclassified variants on splicing or to verify the disease gene by functional complementation of the cellular phenotype with a construct expressing a wild type copy of the suspected gene [35–37]. Finally, if no mutations can be de detected, growing cells can be used to reconfirm the diagnosis FA by checking MMC sensitivity in cell growth or G2-arrest assays [38, 39].

### 2.2. Mutation Screening Strategy

#### 2.2.1. Hints from Ethnic Background or Phenotype

Information on the ethnic background of the proband may provide a clue for a specific pathogenic mutation that most likely causes the disease, such as c.711 + 4A > T (IVS4 + 4A > T) in *FANCC,* a mutation present in homozygous state in 80% of all FA cases of Ashkenazi Jewish ancestry, and c.295C > T in *FANCA*, which was present homozygously in all 40 FA cases of Spanish Gypsy ancestry so far investigated. More examples of recurrent mutations are shown in Table 3. The distinct clinical phenotype of D1 and N patients (severely affected, often combined with leukemia or solid tumors below the age of 5 years) may provide a clue to favor *BRCA2/FANCD1 *and *PALB2/FANCN* as the first gene to be screened [40–44]. This is especially worthwhile if confirmed by the cellular phenotype: in contrast to cells from all other known FA subtypes, cells from D1, N and O patients are unable to form RAD51 foci upon exposure to X rays or MMC [43–45].

#### 2.2.2. No Clues Available

1. In the absence of any clue to the disease gene, mutation screening starts with a search for deletions in *FANCA*, as this type of mutation accounts for 40% of all mutant *FANCA* alleles. The quantitative multiplex ligation-dependent probe amplification (MLPA) method [46] is used for this initial screen, which identifies *FANCA* as the most likely disease gene in 1 out of 4 patients by the detection of a—usually hemizygous-deletion. In parallel, the *FANCA* gene is completely sequenced. The combination of these two approaches identifies 60–70% of all FA patients as FA-A.
2. Next, *FANCC, -E, -F*, and *-G* are screened by DNA sequencing.
3. Only if the proband is a male, *FANCB* is screened by MLPA and DNA sequencing,

In Table 4, optimized conditions are provided for the PCR amplification of *FANCA, -C, -E, -F, -G, and -B*. Most PCRs can be performed under standard conditions. The PCR primers have M13 extensions which allow sequencing of all fragments with universal sequencing primers. MLPA was performed according to the instructions of the supplier. Detailed information about the sequences of the MLPA probes is available from the website of the supplier (http://www.mlpa.com). In a well-equipped laboratory with sufficient dedicated personal, testing of *FANCA, -C, -E, -F, -G and -B* can be completed within 1-2 weeks.

After screening *FANCA, -C, -E, -F, -G, and –B,* a molecular diagnosis is obtained for ~85% of the patients [34]. In our cohort of 54 patients, referred to our diagnostic service since 2008, mutations were detected in 45 patients (83%). *FANCA* mutations were found in 31 of the patients (57%), *FANCC* mutations in 6 patients (11%), and *FANCG* mutations in 5 patients (9%). *FANCB*, *FANCE,* and *FANCF* mutations were found in single families (Table 3). In the small group of patients without mutations no complementation analysis or FANCD2 western blotting was performed. Therefore, we do not know if we missed *FANCA, -C, -E, -F, -G, and -B* mutations in these patients or that these patients have mutations in other FA genes. Table 3 does not include prenatal cases, because prenatal testing is only offered in couples in which the FA-causing mutations are already established. Testing was offered as a diagnostic service for which a fee was charged.

For the patients negative for *FANCA, -C, -E, -F, -G, and -B* mutations, next generation sequencing can be used to analyze all other FA genes. If this technique is not available, further analysis will depend on the availability of growing cells from the proband. In that case a western blot should reveal whether both FANCD2 isoforms are present at normal levels.

1. If both FANCD2 bands are absent or very weak, *FANCD2 *is sequenced. Because of the presence of *FANCD2* pseudogene sequences in the genome, this testing must be performed on cDNA or gDNA using specially designed primers [26].
2. If only the short isoform of FANCD2 is present, *FANCL* and *FANCM* are sequenced. If no mutations are found, the patient may be mutated in *FANCI* or in another unidentified FA gene acting upstream of FANCD2.
3. If both isoforms are present, and if the clinical phenotype is compatible with FA-D1 or FA-N, *BRCA2/FANCD1* and *PALB2/FANCN* are screened by MLPA and DNA sequencing.
4. If negative, *BRIP1/FANCJ*, *PALB2/FANCN*, *RAD51C/FANCO,* and *SLX4/FANCP* are sequenced.
5. If negative again, the patient should be screened for mutations in *NBS1*, *ESCO2* and *DDX11* to test for Nijmegen breakage syndrome, Roberts syndrome and Warsaw Breakage syndrome, respectively [47, 48]. The latter two syndromes can also be excluded by analyzing metaphase spreads for sister chromatid cohesion defects. If again negative, the patient is likely to be mutated in a novel FA gene acting downstream of FANCD2 ubiquitination.

## 3. Notes

### 3.1. Mutation Screening in Mosaic Patients

If an available lymphoblastoid cell line from an FA patient is phenotypically normal due to genetic reversion at the disease locus, mutation screening is still possible in the reverted cell line, since at least one mutation will be present [49–51]. The second mutation may be identified through investigating the parents.

### 3.2. Unclassified Variants

Missense mutations or *in-frame* deletions or insertions should be judged using *in silico* prediction algorithms (SIFT, POLYPHEN2, Align GVGD). Alternatively, they can be tested for pathogenicity in a cellular transfection assay to check the ability of the variant gene product to complement the cellular FA defect in a deficient cell line (see e.g., [10, 35, 52]). Generally, these tests are only feasible in a setting where a diagnostic laboratory is equipped with a research laboratory with all necessary technology.

### 3.3. Functional Assignment to Genetic Subtypes

 Retroviral constructs have been used to identify the FA subtype by functional complementation, as an intermediate step before a mutation screen is undertaken [36]. Although knowing the disease gene facilitates mutation screening, retroviral transduction has some drawbacks in comparison to direct mutation screening: (i) growing, MMC-sensitive cells either from a cell line or fresh blood sample are required, which are not always easy to obtain; (ii) overexpression of some FA proteins (e.g., FANCM and FANCP) may be toxic for cells; (iii) novel genetic subtypes that emerge after all known groups have been excluded and cannot be readily distinguished from false negatives, that is, transductions that for some unknown reason have failed to cause complementation; (iv) the method requires relatively advanced laboratory facilities and technology. However, functional assignment of complementation group can rapidly be provided by laboratories with capability for this type of analysis [37], which has greatly facilitated reliable genotyping for over 95% of FA patients for which viral constructs are available.

### 3.4. Genetic Counseling

All patients with a diagnosis of FA confirmed by mutation analysis should be referred for genetic counselling, together with their parents and siblings. Mutation testing should be performed in all sibs regardless of any clinical symptoms. A complete pedigree, including a cancer history anamnesis, should be prepared. Mutation carriers might be at increased cancer risk (see Section 3.7) whose aspect should be included in the counseling (see Section 3.7). 

FA patients themselves usually have decreased fertility. Women usually have late menarche, irregular menses, and early menopause. However, pregnancies in women with FA have been described, and therefore women should be adequately informed about the risks for their offspring, which is mainly related to an increase in pregnancy-related complications [53].

Sibs of the parents of an FA patient often request carrier screening to assess their risk of getting a child with FA. If a sib appears to be carrier, this risk is still minimal because of the very low carrier frequency in the population. In the US the carrier frequency has been estimated to be about 1 in 181 [54]. The risk of a proven carrier to get a child with FA is therefore about 1 in 724. However, in small communities or in consanguineous couples this risk is much higher, and mutation screening in spouses of proven carriers may be indicated.

### 3.5. Prenatal Diagnosis

Prenatal diagnosis of FA is relatively straightforward after the pathogenic mutations in a given family have been identified. Fetal cells can be obtained by chorionic villus sampling (CVS) during weeks 10–12 of the pregnancy or by amniocentesis, which is performed between weeks 14 and 16. However, CVS may be preferred as the diagnosis will be known at an earlier stage. If the mutation is not known, a chromosomal breakage test on fetal material may be performed [55], but this test may be considered less reliable than screening for mutations in the fetal material. Alternatively, flow cytometric testing of MMC sensitivity in amniotic cell cultures might be an option; however this technique is only available in a limited number of specialized laboratories [56]. Occasionally, FA may be suspected by fetal ultrasound imaging and confirmed by parental carrier testing when the family is not yet known to carry a risk for FA [57].

### 3.6. Genotype-Phenotype Correlation

FA is considered as one disease, and the question may be raised whether all fifteen genetic subtypes equally conform to the clinical FA phenotype. Genotype-phenotype correlation studies comparing the 3 most common groups A, C, and G indicated modest phenotypic differences, which were rather correlated with the relative severity of the mutations [23]. However, bias due to the ethnic distribution of the studied population is very well possible. Other studies reported significant differences between FA-A/G versus FA-C [58]. Cases in group FA-D1 (mutated in BRCA2) and FA-N (mutated in *PALB2*) present with a distinct, relatively severe, phenotype that is characterized by the development of leukemia at very young age (median 2.2 years) and by pediatric cancers such as nephroblastoma (Wilms tumor) or medulloblastoma [40–44]. The observations that one of the pathogenic mutations in *BRCA2* in FA-D1 patients is hypomorphic and that mice with biallelic null alleles in *Brca2* are embryonic lethals suggest that the BRCA2 protein serves a function that is essential for survival.

Different mutations in the same gene may be associated with divergent phenotypes, as illustrated by the two *FANCC* mutations, c.711+4A>T and c.67delG. The former (splice-site) mutation is associated with a relatively severe phenotype in Ashkenazi Jewish people [19] although the associated phenotype was reportedly less severe in patients of Japanese ancestry [20]. The carrier frequency for this mutation in the Ashkenazi population is relatively high (1 in 87), which has led to the recommendation of carrier detection to prevent disease [59]. In the Netherlands more than 50% of FA cases are homozygous for the *FANCC* frameshift mutation c.67delG. The phenotype associated with this mutation, like other exon 1 mutations, seems relatively mild, as these patients rarely have skeletal abnormalities and show a relatively late age of onset of their marrow failure [24]. Awareness of such genetically determined phenotypic differences may help in clinical decision making, including the counselling of patients and families.

### 3.7. Cancer Risk in Heterozygous Mutation Carriers

An important issue is whether FA mutation carriers are at increased risk to develop cancer or other types of disease. Overall, there is no increased risk for cancer among FA heterozygotes [60, 61]. However, the situation is different in some of the less prevalent FA subtypes. The FA-D1 subtype is caused by mutations in *BRCA2* [62] which is a well-known breast and ovarian cancer predisposition gene [63]. In FA-D1 one of the mutations will be hypomorphic because biallelic “severe” mutations are supposed to be lethal [26]. Therefore, one of the parents of a FA-D1 patient will be a heterozygous carrier of a “severe” inactivating *BRCA2 *mutation and may thus have an increased risk for breast cancer and other BRCA2-associated cancers. Whether the parent with the hypomorphic mutation is also at increased risk is unknown: in breast cancer families these hypomorphic mutations are considered as variants with unknown clinical significance. Two other genes involved in FA and related to breast or ovarian cancer predisposition are *PALB2/FANCN* [64, 65] and *RAD51C/FANCO* [66]. Although cancer patients have been identified with germ-line mutations in these genes, an accurate estimate of the relative cancer risk for mutation carriers is still lacking.

Another special case is represented by female *FANCB* mutation carriers, who are supposed to consist of 50% FA-like cells due to silenced expression of the wild type *FANCB* allele by the random process of X inactivation that occurs during early embryonic development. Nevertheless, in the few female *FANCB* mutation carriers studied so far, inactivation appeared strongly skewed towards the mutated allele [67]. This suggests that FA cells have a poor chance to survive next to unaffected cells in the same tissue, and these FA cells may therefore not give an increased cancer risk. However, the data are scarce at present so that no firm conclusions can be drawn regarding the cancer risk of female *FANCB* mutation carriers [60].

## Figures and Tables

**Table 1:** Fanconi anemia complementation groups, genes, and proteins.

Group	Gene symbol(s)^a^	Cytogenetic location	Protein (amino acids)	Domain structure (references)
A	*FANCA*	16q24.3	1455	HEAT repeats [8]
B	*FANCB*	Xp22.31	859	—
C	*FANCC*	9q22.3	558	HEAT repeats [8]
**D1** ^ b^	*BRCA2*	13q12.3	3418	RAD51- and DNA-binding motifs [9]
D2	*FANCD2*	3p25.3	1451	—
E	*FANCE*	6p21.3	536	—
F	*FANCF*	11p15	374	—
G	*FANCG*	9p13	622	Tetratricopeptide repeats (TPR) [10]
I	*FANCI*	15q26.1	1328	—
**J** ^b^	*BRIP1*	17q22	1249	DNA helicase [11, 12]
L	*FANCL*	2p16.1	375	RING finger motif (E3 ligase) [7, 8]
M	*FANCM*	14q21.3	2014	DNA helicase, nuclease [13]
**N** ^b^	*PALB2*	16p12.1	1186	—
**O** ^b^	*RAD51C*	17q25.1	376	—
**P** ^b^	*SLX4*	16p13.3	1834	Endonuclease scaffold [3, 4]

^a^For gene nomenclature see http://www.genenames.org/.

^b^The proteins defective in groups D1, J, N, O, and P (boldface) act downstream or independent of the monoubiquitination of FANCD2; all other FA proteins act upstream of this process.

**Table 2:** Major recurrent mutations in FA.

Gene	Mutation*	Geographic/ethnic background	Comment	References
*FANCA*	c.3788_3790del (p.Phe1263del)	European, Brazilian	Relatively mild	[14, 15]
	c.1115_1118delTTGG (p.Val372fs)	European	Relatively mild	[16]
	Exon 12–17del Exon 12–31del	South-African	Relatively common in Afrikaners	[17]
	c.295C>T (p.Gln99X)	Spanish Gypsy population	Worldwide highest prevalence of mutant *FANCA* allele	[18]
*FANCC*	c.711+4A>T (originally reported as IVS4+4A>T)	Homozygous in 80% of Ashkenazi Jewish FA; relatively common in Japan.	Severe phenotype in Jews, milder in Japanese.	[19–22]
	c.67delG (originally reported as 322delG)	Homozygous in approx. 50% of Dutch FA patients	Like other exon 1 mutations, relatively mild phenotype.	[19, 23–25]
*FANCD2*	c.1948-16T>G	Turkish	Founder mutation	[26]
*FANCG*	c.313G>T (p.Glu105X)	European	44% of mutated *FANCG* alleles in Germany.	[27]
	c.1077-2A>G	Portuguese/Brazilian	Founder mutation	[27, 28]
	c.1480+1G>C	French-Canadian	Founder mutation	[28]
	c.307+1G>C	Japanese	Founder mutation	[28, 29]
	c.1794_1803del (p.Trp599fs)	European		[28]
	c.637_643del (p.Tyr213fs)	Sub-Saharan Africa	82% of all black FA patients	[30]
*FANCJ*	c.2392C>T (p.Arg798X)		Found in ca. 50% of FA-J patients of diverse ancestry; ancient mutation or hot spot.	[11, 12]

Nucleotide numbering based on ATG = +1.

Published sequence variations in FA genes, with their descriptions conforming to the current nomenclature rules, are listed at http://www.rockefeller.edu/fanconi/.

**Table 3:** Mutations detected in a cohort of 54 patients by screening *FANCA, FANCC, FANCE, FANCF* and *FANCG. *

			Allele 1			Allele 2		
	Country of origin^1^	Gene	DNA change	Effect	Number of database entries	DNA change	Effect	Number of database entries
1	ES	FANCA	ex16_17del	del	12x	c.1115_1118del	p.Val372fs	62x
2	PT	FANCA	c.718C>T	p.Gln240X	2x	c.2870G>A	W957X	1x
3	NL	FANCA	ex15del	del	3x	ex15del	del	3x
4	NL	FANCA	c.3788_3790del	p.Phe1263del	215x	c.3788_3790del	p.Phe1263del	215x
5	CA	FANCA	c.718C>T	pGlnx240X	2x	c.1085T>C	p.Leu362Pro	novel
6	PT	FANCA	c.3788_3790del	p.Phe1263del	215x	c.4130C>G	p.Ser1377X	1x
7	IE	FANCA	c.2812_2830dup	p.Asp944fs	3x	c.2812_2830dup	p.Asp944fs	3x
8	AU	FANCA	c.2303T>C	p.Leu768Pro	5x	c.2303T>C	p.Leu768Pro	5x
9	NL	FANCA	c.862G>T	p.Glu288X	1x	c.862G>T	p.Glu288X	1x
10	NL	FANCA	ex11_33del	del	1x	c.2121delC	p.Asn707fs	novel
11	DK	FANCA	ex1_8del	del	1x	c.3788_3790del	p.Phe1263del	215x
12	UK	FANCA	c.337_338del	p.Ala114fs	1x	c.3349A>G	p.Arg1117Gly	2x
13	UK	FANCA	c.3568C>T	p.Gln1190X	novel	c.3568C>T	p.Gln1190X	novel
14	NL	FANCA	c.487delC	p.Arg163fs	1x	c.2851C>T	p.Arg951Trp	11x
15	SE	FANCA	c.88delG	p.Val30fs	novel	c.100A>T	p.Lys34X	2x
16	NL	FANCA	c.862G>T	p.Glu288X	9x	c.1771C>T	p.Arg591X	9x
17	PT	FANCA	c.1709_1715+4del	p.Glu570fs	novel	c.3430C>T	p.Arg1144Trp	novel
18	NO	FANCA	c.100A>T	p.Lys34X	2x	c.1378C>T	p.Arg460X	novel
19	PT	FANCA	ex15_17del	del	2x	ex15_17del	del	2x
20	NL	FANCA	c.2982-192A>G	splice^2^	novel	ex7_31del	del	
21	AU	FANCA	c.427-8_427-5del	splice	novel	c.1771C>T	p.Arg591X	9x
22	AU	FANCA	c.3491C>T	p.Pro1164Leu	novel	c.3491C>T	p.Pro1164Leu	novel
23	CA	FANCA	ex4_29del	del	novel	ex31del	del	6x
24	NL	FANCA	c.3391A>G	p.Thr1131Ala	15x	c.3391A>G	p.Thr1131Ala	15x
25	GR	FANCA	c.2T>C	p.Met1?	1x	c.3788_3790del	p.Phe1263del	215x
26	IE	FANCA	c.851dup	p.Val285fs	novel	c.2534T>C	p.Leu845Pro	4x
27	NL	FANCA	c.2852G>A	p.Arg951Gln	6x	c.3624C>T	p.= (splice)	2x
28	AU	FANCA	c.331_334dup	p.Leu112fs	novel	ex22_29del	del	novel
29	NL	FANCA	c.862G>T	p.Glu288X	9x	c.3920delA	p.Gln1307fs	2x
30	IR	FANCA	ex21del	del	novel	ex21del	del	novel
31	SE	FANCA	ex1_12del	del	novel	ex22_29del	del	novel
32	NL	FANCB	c.755_767del	p.Leu252fs	novel	—	—	—
33	NL	FANCC	c.67delG	p.Asp23fs	50x	c.553C>T	p.Arg185X	14x
34	NL	FANCC	c.67delG	p.Asp23fs	50x	c.67delG	p.Asp23fs	50x
35	CA	FANCC	c.67delG	p.Asp23fs	50x	c.553C>T	p.Arg185X	14x
36	NL	FANCC	c.67delG	p.Asp23fs	50x	c.1155-1G>C	splice	novel
37	NL	FANCC	c.67delG	p.Asp23fs	50x	c.67delG	p.Asp23fs	50x
38	NL	FANCC	c.67delG	p.Asp23fs	50x	c.467delC	p.Ser156fs	novel
39	PT	FANCE	c.1111C>T	p.Arg371Trp	6x	c.1111C>T	p.Arg371Trp	6x
40	UK	FANCF	c.496C>T	p.Gln166X	4x	c.496C>T	p.Gln166X	4x
41	UK	FANCG	c.307+2delT	splice	novel	c.307+2delT	splice	novel
42	UK	FANCG	c.1471_1473delinsG	p.Lys491fs	novel	c.1471_1473delinsG	p.Lys491fs	novel
43	NL	FANCG	c.65G>C	p.Arg22Pro	6x	c.65G>C;	p.Arg22Pro	6x
44	IR	FANCG	c.307+1G>C	splice	21x	c.307+1G>C	splice	21x
45	NL	FANCG	c.85-1G>A	splice	novel	c.85-1G>A	splice	novel

^1^Country of origins: AU: Australia; CA: Canada; DK: Denmark; ES: Spain; GR: Greece; IE: Ireland; IR: Iran; NL: Netherlands; PT: Portugal; SE: Sweden; UK: United Kingdom

Number of database entries refers to the FA database at: http://www.rockefeller.edu/fanconi/.

The pathogenic state of novel missense mutations is based upon *in silico* prediction algorithms (SIFT, POLYPHEN2, Align GVGD), the presence of a second clearly pathogenic mutation in the same gene and segregation in the family.

^2^Effect c.2982-192A>G: by studying cDNA it was shown that the mutation created a new splice donor site resulting in an aberrant mRNA.

**Table:** (a)

*FANCA*		
Primer name	Sequence (5′ > 3′)	Product length (bp)
FANCA_ex1F	gtaaaacgacggccag GCGCCTCCCCCAGGACCAACA	362
FANCA_ex1R	caggaaacagctatga AGGCTCTGGCGGGAAGGGATCGG	
FANCA_ex2F	gtaaaacgacggccag CTCTTCGGGAGGGTGTCGCTGGT	328
FANCA_ex2R	caggaaacagctatga CTCTTCGGGAGGGTGTCGCTGGT	
FANCA_ex3F	gtaaaacgacggccag GCCTGGCCTGGAGCTTGAAT	392
FANCA_ex3R	caggaaacagctatga CGCAGGTTGAATCAGACGCTGTT	
FANCA_ex4F	gtaaaacgacggccag TAAGGCATTTTAAACAGCAAGTC	430
FANCA_ex4R	caggaaacagctatga TGCCAATAAATACTGAGCAAACT	
FANCA_ex5F	gtaaaacgacggccag AGTATTGTTTCAGGTAATTTGTT	356
FANCA_ex5R	caggaaacagctatga TGAAGGTACTTCTTTCCAATCCA	
FANCA_ex6F	gtaaaacgacggccag AGATGTGTTTCAGGCTCTAAGTT	402
FANCA_ex6R	caggaaacagctatga GCAATGCAATCTAGTCTAGTACA	
FANCA_ex7F	gtaaaacgacggccag TGGGATTTAGTTGAGCCTTACGTCTGC	421
FANCA_ex7R	caggaaacagctatgaAAGGTGAATGGAAACACTTAAACTCATGTCA	
FANCA_ex8F	gtaaaacgacggccag GTGGTCAGGTGAGCAGTAACTTC	401
FANCA_ex8R	caggaaacagctatga TAAATAGGTACAAACAGCACGTT	
FANCA_ex9F	gtaaaacgacggccag TTCTCTTGTGTGATGCAGGTATC	332
FANCA_ex9R	caggaaacagctatga TGACCCACAGATTCATGAGGTAT	
FANCA_ex10F	gtaaaacgacggccag TTTTGATTAAGGCCTACAGATTG	406
FANCA_ex10R	caggaaacagctatga CCTCCTCCTCACGCACGTTATCG	
FANCA_ex11F	gtaaaacgacggccag TTTCAAGTCTGTGGTTATAGTGG	410
FANCA_ex11R	caggaaacagctatga AGACGTAAAAGAGGTCCTAGAAT	
FANCA_ex12F	gtaaaacgacggccag CTGTGGGGCTGGTCCTTAACAAA	236
FANCA_ex12R	caggaaacagctatga AGGCAGCATGACAAGTACTAGGC	
FANCA_ex13F	gtaaaacgacggccag ACATTGGTTTGCTTGGATATTGA	377
FANCA_ex13R	caggaaacagctatga CTGACAAAGAATGTTCCATCGAC	
FANCA_ex14F	gtaaaacgacggccag TGCTGTAATTGCTGTGTAGTCTT	411
FANCA_ex14R	caggaaacagctatga ACTCACATGACAGAGAATCAGGT	
FANCA_ex15F	gtaaaacgacggccag ACTACAGCAGCCGCCCGGACACT	430
FANCA_ex15R	caggaaacagctatga GCAGATCTGCAGGAGGCTCTTGG	
FANCA_ex16F	gtaaaacgacggccag TCCCAGGCAGTTCCCAGACTAAC	312
FANCA_ex16R	caggaaacagctatga AGCTGATGACAAATCCTCGTAGA	
FANCA_ex17F	gtaaaacgacggccag ACCGCTCCCTCCTCACAGACTAC	334
FANCA_ex17R	caggaaacagctatga AAGGCTGAAAAACTCAACTCAAG	
FANCA_ex18F	gtaaaacgacggccag GCGCACAGCATGTGGGCCTTTAC	397
FANCA_ex18R	caggaaacagctatga GCAGCTGCTAGAGGCCTTTTCGG	
FANCA_ex19F	gtaaaacgacggccag GTGCACAAGAAGACTTCATAATG	284
FANCA_ex19R	caggaaacagctatga AGTCCTTGCTTTCTACACAACTG	
FANCA_ex20F	gtaaaacgacggccag CTTCTCTGTGTTGCAGCATATTC	298
FANCA_ex20R	caggaaacagctatga AGAAGAAACCTGGAAGTAGTCAT	
FANCA_ex21F	gtaaaacgacggccag ATAATAGATTTGGGGATTGTAAT	255
FANCA_ex21R	caggaaacagctatga CAACAGACACTCAAGGTTAGGAA	
FANCA_ex22F	gtaaaacgacggccag TGCAGTGAAGAGTCCTGTTGAGT	305
FANCA_ex22R	caggaaacagctatga ACACACCAGCCTGATGTCACTAT	
FANCA_ex23F	gtaaaacgacggccag CAGTCAGCAGGATCCGTGGAATC	416
FANCA_ex23R	caggaaacagctatga GGCCCTGGAACATCTGATACGAC	
FANCA_ex24F	gtaaaacgacggccag CCTTCCTGCTGCTCCCGTCC	229
FANCA_ex24R	caggaaacagctatga CAGACTTGGCCCAGCAAGAG	
FANCA_ex25F	gtaaaacgacggccag CCGCTGGTGGTTGGATTAGCTGT	296
FANCA_ex25R	caggaaacagctatga TTTCCAGGGCACTGAAGACGAAT	
FANCA_ex26F	gtaaaacgacggccag AGCTTGGAAGAGGGCAGTCTGCT	347
FANCA_ex26R	caggaaacagctatga CTCTTCTAATTTTATCAAACGAG	
FANCA_ex27F	gtaaaacgacggccag AGACTGTCTCACAACAAACGAAC	356
FANCA_ex27R	caggaaacagctatga CGGTCCGAAAGCTGCGTAAAC	
FANCA_ex28F	gtaaaacgacggccag GTTGATGGTCTGTTTCCACCTGA	401
FANCA_ex28R	caggaaacagctatga GAAGGAACGGTCACCTACGTGCT	
FANCA_ex29F	gtaaaacgacggccag GACATGGAGGACTGCGTATGAGA	411
FANCA_ex29R	caggaaacagctatga GTGGCTGTGATGACTGGAACGTG	
FANCA_ex30F	gtaaaacgacggccag CCCGAGCCGCCAGTCTCAACCCA	411
FANCA_ex30R	caggaaacagctatga AAAGGCAGACCCACCCTAAGCTA	
FANCA_ex31F	gtaaaacgacggccag GATAAGCCTCCTTGGTCATGGTA	406
FANCA_ex31R	caggaaacagctatga TGGCAATAAATATCTTAATAGCA	
FANCA_ex32F	gtaaaacgacggccag TTCCTGTGCCAGCATACTGCTCT	359
FANCA_ex32R	caggaaacagctatga GGGTGGGGACACACAGACAAGTA	
FANCA_ex33F	gtaaaacgacggccag TGGGTTTCAGGGTGGTGGTTGCT	356
FANCA_ex33R	caggaaacagctatga GAACCCTTTCCTCAGTAATTCAC	
FANCA_ex34F	gtaaaacgacggccag CGCCCAGGGAAGCCGTTAAGTTT	333
FANCA_ex34R	caggaaacagctatga GCGTTCTGAGAAGGCCACGAGAG	
FANCA_ex35F	gtaaaacgacggccag TTCCTTCACTCTACTAGTTGTGG	311
FANCA_ex35R	caggaaacagctatga TGAGATGGTAACACCCGTGATGG	
FANCA_ex36F	gtaaaacgacggccag CCATCTCAGCCACCCTCATCTGT	350
FANCA_ex36R	caggaaacagctatga AGGCGCCCACCACCACGAGAACT	
FANCA_ex37F	gtaaaacgacggccag GACTTGGTTTCTATGGCGTGGTT	310
FANCA_ex37R	caggaaacagctatga CCCAGAGAAATAGCACTGATTGA	
FANCA_ex38F	gtaaaacgacggccag GTTTTCTAAGATCCACTTAAAGG	362
FANCA_ex38R	caggaaacagctatga CTCACTCACACTTCCGCAAACAC	
FANCA_ex39F	gtaaaacgacggccag CTGTCCAGAGGCCCAGTATTACC	387
FANCA_ex39R	caggaaacagctatga AGGAGGGCTCGTTCTTAACCATT	
FANCA_ex40F	gtaaaacgacggccag GGTGTCCCCAGCACTGATAATAG	353
FANCA_ex40R	caggaaacagctatga AGACATAGTGACAAATGGCTACA	
FANCA_ex41F	gtaaaacgacggccag CCCTTGGCATCACCTGCTACCTT	403
FANCA_ex41R	caggaaacagctatga AACAGGCAAACTCACAGGTTAGA	
FANCA_ex42F	gtaaaacgacggccag ACCAGCCCTGTTTCTGTATGTCT	248
FANCA_ex42R	caggaaacagctatga ACATGGCCCAGGCAGCTGTCAAT	
FANCA_ex43F	gtaaaacgacggccag TGTGGGGGACATGAGAATTGACA	378
FANCA_ex43R	caggaaacagctatga GTAATCCACTTTTTAGTGCAACA	
FANCAIVS10F	gtaaaacgacggccag TTTACATGTGCATCAGTTAGCTT	184
FANCAIVS10R	caggaaacagctatga CATGAAGACACAGAAAAAGTAGGT	

**Table:** (b)

*FANCC*		
Primer name	Sequence (5′ > 3′)	Product length (bp)
FANCC_ex1F FANCC_ex1R	gtaaaacgacggccag ACCATTTCCTTCAGTGCTGGACA caggaaacagctatga CCATCGGCACTTCAGTCAATACC	378
FANCC_ex2F FANCC_ex2R	gtaaaacgacggccag CTAAACAAGAAGCATTCACGTTC caggaaacagctatga GGAGAAAGGTTCATAATGTAAGC	303
FANCC_ex3F FANCC_ex3R	gtaaaacgacggccag TCAGCAGAAAGAGAATGTGCAAA caggaaacagctatga AACATCATAGAACTGGATTCCAC	405
FANCC_ex4F FANCC_ex4R	gtaaaacgacggccag TGTACATAAAAGGCACTTGCATT caggaaacagctatga TCCCATCTCACATTTCTTCCGTA	380
FANCC_ex5F FANCC_ex5R	gtaaaacgacggccag AGAACTGATGTAATCCTGTTTGC caggaaacagctatga TTACTGCTCTGTGAGAGTTGAGA	367
FANCC_ex6F FANCC_ex6R	gtaaaacgacggccag GTCTTTGACCTTTTTAGCATGAA caggaaacagctatga AACGTTTGGACACTGCTGTCGTA	387
FANCC_ex7F FANCC_ex7R	gtaaaacgacggccag ATTAGTGATTGCATTTTGAACTT caggaaacagctatga CAAAAATAAAATGTAAATACACG	422
FANCC_ex8F FANCC_ex8R	gtaaaacgacggccag CTCCTTTGGCTGATAATAGCAAG caggaaacagctatga CTGATTTTTGAGTTTTTACCTCT	336
FANCC_ex9F FANCC_ex9R	gtaaaacgacggccag ATACTGCTGAAGCTTATGGCACA caggaaacagctatga TAACCTTTGTTGGGGCACTCATT	400
FANCC_ex10F FANCC_ex10R	gtaaaacgacggccag TATGAGGTTATTGGGAGCTTATT caggaaacagctatga CTGTCTCCCTCATGCTGTAGATA	382
FANCC_ex11F FANCC_ex11R	gtaaaacgacggccag GAACCAGAAGTAAAGGGCGTCTC caggaaacagctatga CTGACCTGCTCCAAGCCATCCGT	416
FANCC_ex12F FANCC_ex12R	gtaaaacgacggccag AAGTACAATTTAAGCCAACCGTT caggaaacagctatga AGGTTGCCATGACATATGCCATC	451
FANCC_ex13F FANCC_ex13R	gtaaaacgacggccag CCTCTCTCAGGGGCCAGTGCTTA caggaaacagctatga AGACCCTCGGACAGGTAACCCAC	435
FANCC_ex14F FANCC_ex14R	gtaaaacgacggccag ACTTGCTATGCTAATCACCTTGC caggaaacagctatga AATGCGTGGCCACAGGTCATCAC	437

**Table:** (c)

*FANCE*		
Primer name	Sequence (5′ > 3′)	Product length (bp)
FANCE_ex1F FANCE_ex1R	gtaaaacgacggccag CGCCTCCCTCCTTCCCTTTC caggaaacagctatga CCCGCCTCCCATACCTGCTAA	540
FANCE_ex2aF FANCE_ex2aR	gtaaaacgacggccag GCTCTGCCCAGTCTGCCTTGTGC caggaaacagctatga CTCTGAGTCCTTTCTGCGTTTCC	469
FANCE_ex2bF FANCE_ex2bR	gtaaaacgacggccag GCCAGAGACAGCTCCAAAGTCTA caggaaacagctatga CAGCCTTCCCCATGGATAAAGCC	479
FANCE_ex3F FANCE_ex3R	gtaaaacgacggccag GCCTCTTGACTTTCTTGAATCAT caggaaacagctatga ACTGTCCTCAGACCTTTACTCCA	352
FANCE_ex4F FANCE_ex4R	gtaaaacgacggccag TTGAACCAAGTGTAGACTTACCA caggaaacagctatga GGGAAGGAACCAAGGGCTAAAAG	436
FANCE_ex5F FANCE_ex5R	gtaaaacgacggccag GTATCTTTTAGCCCTTGGTTCCT caggaaacagctatga GAATCCCCTCTCTCAAGTACCAC	431
FANCE_ex6F FANCE_ex6R	gtaaaacgacggccag TTTCCTTTGTAACATGTATCATC caggaaacagctatga AGCAGAAAGCAGGGAGGCGGTAA	433
FANCE_ex7F FANCE_ex7R	gtaaaacgacggccag ACAGGCTGGGCATTCTGTTACCG caggaaacagctatga AGTGAGACACAAGGATCCCCTAA	425
FANCE_ex8F FANCE_ex8R	gtaaaacgacggccag TTGGAGCAGCAGATAGATACTCA caggaaacagctatga AGAGGTGGAGCTGAAGTGACCAT	380
FANCE_ex9F FANCE_ex9R	gtaaaacgacggccag GTTACCTGCCCAGGGTCACCTAG caggaaacagctatga CTGGCCAGCACTCAGGGTTTTAT	388
FANCE_ex10F FANCE_ex10R	gtaaaacgacggccag TGGCCTCCTCTCTCCTCAATAGA caggaaacagctatga AACAGGGAGGCAGTTGCAATCTG	369

**Table:** (d)

*FANCF*		
Primer name	Sequence (5′ > 3′)	Product length (bp)
FANCF_ex1aF FANCF_ex1aR	gtaaaacgacggccag TTTCGCGGATGTTCCAATCAGTA caggaaacagctatga CTGCACCAGGTGGTAACGAGCTG	449
FANCF_ex1bF FANCF_ex1bR	gtaaaacgacggccag AGTGGAGGCAAGAGGGCGGCTTT caggaaacagctatga GCTATCACCTTCAGGAAGTTGTT	456
FANCF_ex1cF FANCF_ex1cR	gtaaaacgacggccag CCCAAATCTCCAGGAGGACTCTC caggaaacagctatga TTTCTGAAGGTCATAGTGCAAAC	444
FANCF_ex1dF FANCF_ex1dR	gtaaaacgacggccag GCTTTTGACTTTAGTGACTAGCC caggaaacagctatga ATTTGGTGAGAACATTGTAATTT	456

**Table:** (e)

*FANCG*		
Primer name	Sequence (5′ > 3′)	Product length (bp)
FANCG_ex1F FANCG_ex1R	gtaaaacgacggccag AGCCTGGGCGGGTGGATTGGGAC caggaaacagctatga TCATTTCTGGCTCTTTGGTCAAG	389
FANCG_ex2F FANCG_ex2R	gtaaaacgacggccag CAGGCCAAGGTAACACGGTTGCT caggaaacagctatga CCAGTCTCCTCTGTGCCTTAAAC	460
FANCG_ex3F FANCG_ex3R	gtaaaacgacggccag TATTGTAGCTGTTTTGGTTGGAG caggaaacagctatga GGTGACAGATGTTGTTTATCCTC	362
FANCG_ex4F FANCG_ex4R	gtaaaacgacggccag GGAGATGGAGGATGAGGTGCTAC caggaaacagctatga CGACCACCAACCCAGCCGCCTGT	411
FANCG_ex5F FANCG_ex5R	gtaaaacgacggccag AGATGGAGATAGGAGAAGACGAG caggaaacagctatga GCTTCATGAAGGCTGCTTAGTGC	454
FANCG_ex6F FANCG_ex6R	gtaaaacgacggccag CAGTTCCATGGGCTTCTTAGACC caggaaacagctatga TCAGGGCTGCAACCAAGTACAAC	393
FANCG_ex7F FANCG_ex7R	gtaaaacgacggccag GCACTGGGGTCCTGTCACCGTAA caggaaacagctatga ATAATCTTTGGGAGCCATACTTC	418
FANCG_ex8F FANCG_ex8R	gtaaaacgacggccag GCTTGTGATGGGGTGACTTGACT caggaaacagctatga AGTTCAGGTCTAGAAGCAAGGTA	438
FANCG_ex9F FANCG_ex9R	gtaaaacgacggccag CCTCCTCAGGGCCCATGAACATC caggaaacagctatga GCAGTGTCTTGAAAGGCATGAGC	400
FANCG_ex10F FANCG_ex10R	gtaaaacgacggccag CAGGACTCTGCATGGTACCAG caggaaacagctatga CCAATCAGAAAATCATCCCTC	460
FANCG_ex11F FANCG_ex11R	gtaaaacgacggccag AGCTCCATGTTCACCTACTTACC caggaaacagctatga CAGTGCCGCATCTGACTTACATC	397
FANCG_ex12F FANCG_ex12R	gtaaaacgacggccag AGGATTTGGGGTTTTGGTGACTG caggaaacagctatga AACTCTTGGGAGCCCTGCATACA	445
FANCG_ex13F FANCG_ex13R	gtaaaacgacggccag CCGCTTCCATATGTGAGTGTAGG caggaaacagctatgaC CACAATAGGTCCAAGGACTCTA	340
FANCG_ex14F FANCG_ex14R	gtaaaacgacggccag CCAAACTAAGGGGTCACATGAAG caggaaacagctatga GATGGTGAAGCAGAAAGCCCTCC	405

**Table:** (f)

*FANCB*		
Primer name	Sequence (5′ > 3′)	Product length (bp)
FANCB_ex3AF FANCB_ex3AR	gtaaaacgacggccag GATATGGTTATTTGAATTCTTAGCAcaggaaacagctatga GCCATCCTTCATCTCATAGCCTAGT	721
FANCB_ex3BF FANCB_ex3BR	gtaaaacgacggccag ATTAACCTCCCTTACATTGTGATAGcaggaaacagctatga CAATAAGACTCCAGAATGAACTCTA	811
FANCB_ex4F FANCB_ex4R	gtaaaacgacggccag TTTACAAATGACAACTACATGAcaggaaacagctatga TTAAGTATAAAACCACCAATAT	391
FANCB_ex5F FANCB_ex5R	gtaaaacgacggccag ACTGCATCTGGCCTATAGTTcaggaaacagctatga AATACCATTTTTACCCAAGC	411
FANCB_ex6F FANCB_ex6R	gtaaaacgacggccag GTATTTCCTGAATTATTGGTATGTC caggaaacagctatga CATAAAAGTCCACCATTATAACCTC	395
FANCB_ex7F FANCB_ex7R	gtaaaacgacggccag TGTTTGGGCCATAAGCCCTA caggaaacagctatga TTCTGGAGCATCAAGACAGT	355
FANCB_ex8F FANCB_ex8R	gtaaaacgacggccag GTTGTTTGTATGACATTTAATCATC caggaaacagctatga ATCATTAAACTCTGCCCATTATCAG	636
FANCB_ex9F FANCB_ex9R	gtaaaacgacggccag AGGTAATTTTGTTGGCACTT caggaaacagctatga ATGCGTTCATTCATGCTAGG	531
FANCB_ex10F FANCB_ex10R	gtaaaacgacggccag AATTGGTTCTGTTTATCATTATGGT caggaaacagctatga CTACTACAGTAAGCCTCGGTGTTTA	686

PCR conditions:

PCR was performed in Applied Biosystems PE9700 system using 96-well plates. PCR reactions (final volume 25 *μ*l) contained 0.5 units Platinum Taq polymerase (Invitrogen), 1,5 mM MgCl_2_, 0.2 mM NTPs (Invitrogen), and 10 pmol primer.

For the large majority of amplicons, standard PCR conditions were used: preheat 95°C, 5 min, denaturation 95°C, 30 sec, annealing 60°C, 30 sec., elongation 72°C, 1 min, number of cycles: 33.

Fragments with a different annealing temperature were *FANCA* exons 5, 7, 13, 21, 26, 31, and 38, *FANCC* exon 7, *FANCF* fragment 1d and *FANCE* exon 1 : 55°C; *FANCA* exon 1 : 64°C. For *FANCE* exon 1 the PCR mix was supplemented with 10% DMSO.

For *FANCB* different PCR conditions were used: preheat 95°C, 5 min, denaturation 95°C, 1 min, annealing 50°C, 1 min., elongation 72°C, 1 min., number of cycles 30. For *FANCB* exon 7 and 9 the annealing temperature was 55°C. For sequencing of exon 7 forward, a special sequencing primer was used: 5′-TTTTTAGAAGGAATGTCTTG-3′.

FA gene specific part of the primer is indicated in capitals. Primers are extended with M13 sequence (indicated in normal letter type), which is used for the sequencing reaction.
